# Predictive biosignature of major depressive disorder derived from physiological measurements of outpatients using machine learning

**DOI:** 10.1038/s41598-023-33359-w

**Published:** 2023-04-25

**Authors:** Nicolas Ricka, Gauthier Pellegrin, Denis A. Fompeyrine, Bertrand Lahutte, Pierre A. Geoffroy

**Affiliations:** 1 MyndBlue, 75008 Paris, France; 2 Psychiatry and Addictology Service, Assistance Publique-Hôpitaux de Paris, GHU Paris Nord, DMU Neurosciences, Hopital Bichat–Claude Bernard, 75018 Paris, France; 3 GHU Paris–Psychiatry & Neurosciences, 1 rue Cabanis, 75014 Paris, France; 4 NeuroDiderot, Inserm, FHU I2-D2, Université de Paris, 75019 Paris, France; 5 CNRS UPR 3212, Institute for Cellular and Integrative Neurosciences, 67000 Strasbourg, France; 6 Psychiatry Department, Bégin Military Hospital, 94160 Saint-Mandé, France

**Keywords:** Depression, Predictive markers, Machine learning

## Abstract

Major Depressive Disorder (MDD) has heterogeneous manifestations, leading to difficulties in predicting the evolution of the disease and in patient's follow-up. We aimed to develop a machine learning algorithm that identifies a biosignature to provide a clinical score of depressive symptoms using individual physiological data. We performed a prospective, multicenter clinical trial where outpatients diagnosed with MDD were enrolled and wore a passive monitoring device constantly for 6 months. A total of 101 physiological measures related to physical activity, heart rate, heart rate variability, breathing rate, and sleep were acquired. For each patient, the algorithm was trained on daily physiological features over the first 3 months as well as corresponding standardized clinical evaluations performed at baseline and months 1, 2 and 3. The ability of the algorithm to predict the patient's clinical state was tested using the data from the remaining 3 months. The algorithm was composed of 3 interconnected steps: label detrending, feature selection, and a regression predicting the detrended labels from the selected features. Across our cohort, the algorithm predicted the daily mood status with 86% accuracy, outperforming the baseline prediction using MADRS alone. These findings suggest the existence of a predictive biosignature of depressive symptoms with at least 62 physiological features involved for each patient. Predicting clinical states through an objective biosignature could lead to a new categorization of MDD phenotypes.

## Introduction

Major depressive disorder (MDD) is a prevalent, disabling, chronic, biologically based disorder that impairs social, occupational, and educational functioning^1^. The World Health Organization estimates that MDD affects 280 million people worldwide^2^. By the age of 65, one in three women and one in five men will have experienced an episode of major depression^3^. MDD is associated with increased suicide risk, morbidity, and mortality and is the leading cause of disability worldwide^2,4^. By 2030, MDD is projected to be the leading cause of disease burden worldwide^5^. Therefore, it is vital to identify MDD as early as possible and to predict how the course of the disease will progress over time.

MDD is characterized by wide variability in disease presentation and response to treatment^6^. Individuals with MDD experience a range of symptoms, which can change over time, and include a persistent state of sadness and hopelessness, anhedonia, fatigue, sleep disturbance, circadian rhythm disruptions, indecision, inability to concentrate and recurrent suicidal ideation^7–9^. Major depressive episodes have a median time to recovery of 2 to 3 years and are highly variable at the individual level. Approximately 30% of patients are resistant to treatment and 60% cycle through treatment discontinuation and resumption; moreover 70% of relapses are not detected in time^10^. This heterogeneity contributes to the difficulty in establishing a diagnosis. Additionally, an MDD diagnosis currently relies solely on subjective markers, (e.g., declarative administered by a clinician, based on the patient narrative) which presents numerous challenges in terms of predicting treatment response, remission, risk of relapse, recovery and, consequently, in prescribing personalized treatments.

Many studies have shown that various physiological measurements are linked to MDD. For instance, predictors of depressive relapse include irregular physical activity, slow motion, physiological changes in breathing and heart rates, and disrupted sleep^11^. Indeed, psychomotor retardation is often considered the cornerstone of MDD^12^, as it reflects a fundamental asthenia of patients intricately associated with avolition. Psychomotor retardation fluctuates over a 24-h period, and manifests especially during the first hours after waking. Evaluation is important in patient follow-up since psychomotor retardation is correlated with the severity of MDD^13^ and its evolution can be considered an indicator of the therapeutic effect^12^. Recording of physical activity (e.g., by actigraphy) can be used to assess psychomotor disturbances in patients with MDD^14–17^. Cardiovascular disorders in patients with MDD may reflect dysregulation of the autonomous nervous system and can manifest as a decrease in heart rate variability^18,19^ (whereas an increase would constitute an indicator of the therapeutic effect)^20,21^, respiratory sinus arrhythmia^22^, or worsening of sympathetic reactivity to stress, which can create paroxysmal reactions responsible for symptoms such as heart palpitations or tachycardia and dyspnea^23^. Sleep is another core element of MDD that influences position in the pathophysiology, phenomenology, history, and evolution of episodes^7,24–28^. Sleep alterations are part of the DSM-5^6^ criteria of MDD and are reported by more than 90% of patients^7,29^. Bidirectional associations have been observed between mood episodes and sleep disturbances^7,24,30^. Sleep alterations worsen depressive symptoms^31^ and are associated with an increased risk of suicide^25^. In terms of objective markers, actigraphy studies have reported alterations in sleep–wake cycles during depressive symptoms with less activity during the daytime and increase wakefulness after sleep onset^32^. Polysomnography (PSG) studies have reported several alterations associated with depression, including a reduced duration of slow wave sleep (SWS), increased duration of rapid eye movement (REM) sleep, reduced REM sleep latency, prolongation of the first REM period, and increased REM density^33–38^.

Predicting the evolution of MDD symptoms using machine learning (ML) is a highly active field of research, although other investigators have mainly focused on the evolution of MDD relative to a specific treatment^39^ or did not determine patient-specific biosignatures^40,41^. A meta-analysis and systematic review^42^ showed that predictive models integrating multiple data types performed better than models with single lower-dimension data types and that ML provides an opportunity to parse clinical heterogeneity and characterize moderators of disease risk and trajectory, both of which aligns with our current work and hypothesis. Interestingly, two recent preliminary studies demonstrated the feasibility of predicting MDD symptoms from longitudinal physiological and behavioral data using wearable devices and ML algorithms^43,44^. Their objective was similar to ours, but their predictive model was not patient-specific, and follow-up of patient evolution was not examined since the data were collected over a shorter period (maximum of 8 weeks). Moreover, a direct comparison between studies using different clinical scales cannot be performed without large sample sizes^45^, which was not the case in the study by Rykov et al.^44^. Nevertheless, the use of ML in psychiatric fields constitutes an unprecedented opportunity to address the symptoms' heterogeneity in individual, monitor disease evolution, and adapt the treatment.

To address the untackled challenges of patient heterogeneity, patient follow-up, and long term forecast of mood status, we propose to take advantage of advances in remote physiological data collection and ML to facilitate patient assessment and follow-up, giving physicians additional time to treat more patients, despite limited resources, which is critical for a disorder whose barriers to effective care include a lack of resources and lack of trained healthcare providers^2^.

The aim of the current work was to develop a novel ML algorithm that can identify the symptoms biosignature and provide a clinical score using physiological data from an individual. Our ML model, the Signature Based Model of Depression (SiBaMoD) is divided into 3 elements: a detrender cancelling out the labels auto-correlation, a feature selection component extracting the patient's biosignature, and a neural network predicting the detrended labels from the selected features. The detrending procedure facilitates learning a clinical score from the data, allowing to get a daily clinical score which, to our knowledge, has never been done in the literature. The feature selection component provides an individual biosignature of depressive symptoms, which is unique to our algorithm.

## Methods

### Study design

The data presented herein are derived from a prospective, multicenter, nonrandomized, open-label clinical trial (ClinicalTrials.gov identifier: NCT05547035) designed specifically for this purpose. Patients diagnosed with MDD were enrolled in the study by their general practitioner or psychiatrist.

The study was designed and conducted in accordance with Good Clinical Practice as defined by the Agence Nationale de Sécurité du Médicament et des Produits de Santé, (ANSM; ID: 2017-A00595-48) and the Declaration of Helsinki. An independent ethics research committee, CPP Sud-Est 1 (ID: 2017-34), approved the protocol and informed consent documents. All patients provided written informed consent prior to participation.

### Inclusion/exclusion criteria

The enrolled patients fulfilled the following inclusion criteria: male or female aged 18 to 65 years; treated for MDD according to the DSM-5 definition; presenting a Montgomery and Åsberg Depression Rating Scale (MADRS) score ≥ 20; French speaker; able to read and write in French; able to understand and follow all study procedures; provided informed consent in writing. Patients were excluded for the following reasons: unable to wear a portable monitor for the study duration (6 months); subject to a severe medical pathology (e.g. neurological, rheumatological) at the investigator’s discretion; resistant depression; chronic depression; dysthymia; depression with psychotic features not congruent with mood, schizophrenia disorder; depression with catatonic features; substance use disorder in the last 6 months; extreme sports during the conduct of the study; pre-existing skin infection at the wearable monitor site; pregnant or lactating woman; participation in another drug or medical device study; inability to give informed consent.

### Study procedures

During the enrollment visit, patients received a portable passive monitoring device (described in section Wearable device and physiological features) that they were asked to wear continuously (i.e., 24 h per day, 7 days a week) for 6 months, except for battery charging and during activities that may represent a risk to the integrity of the device (e.g., showering or participation in contact sports). Charging time of the device was up to 2 h, and patients were instructed to charge it during moments they would not wear it. To minimize data noise, the device was to be worn on the nondominant wrist, a common practice in studies using wearable devices (e.g., actigraphy)^46^. This criterion ensures that reliable features can be derived from raw physiological measurements^47^.

The study period was 6 months and comprised seven periods (baseline and months 1, 2, 3, 4, 5 and 6). At each monthly follow-up visit, physicians assessed the patients’ mood status, which included administration of the MADRS. The clinician administered MADRS^48^ is a widely used and accepted instrument for assessing depression and evaluating treatment efficacy in patients diagnosed with MDD. The Structured Interview Guide for the MADRS (SIGMA) provides structured questions that are be asked exactly as written to ensure that administration of the MADRS is standardized. The interrater reliability of the MADRS according to the intraclass correlation coefficient with the SIGMA has been reported as excellent (r = 0.93)^49^. Appropriate for both clinical and research settings, the MADRS can be used to stratify the severity of depressive symptoms and to evaluate trends in the severity of a patient’s depressive episode and response to treatment. In this study, we stratified the MADRS score^50^: no depression (score: 0–6), mild depression (7–19), moderate depression (20–34), and severe depression (≥ 35).

### Endpoints

The primary endpoint was to compare the change in physiological variable (e.g., motor activity, cardio-respiratory activity, and sleep parameters) with the change in the clinical variable (the MADRS total score) over a period of six months.

The secondary endpoint was to train an algorithm to identify markers of mood disorders using six months of physiological and clinical data.

### Wearable device and physiological features

The wearable device takes the form of a wristband (see Fig. 1 in the Supplementary Materials) and was custom manufactured by Éolane (Angers, France), an ISO 13485-compliant company that adheres to medical standards, including IEC 60601 and EN 62304, for medical device software. The use of a custom device was necessary to obtain the raw measurements from all sensors and to enable the addition of new physiological feature acquisition systems if needed. The custom wearable device contained the following standard sensors: a photoplethysmograph (PPG) with a 50 Hz sampling rate (for deriving cardiorespiratory features), a 3-axis accelerometer with a 25 Hz sampling rate (for deriving multiple actigraphy features) and an electrodermal activity (EDA) sensor with a 4 Hz sampling rate.

**Figure 1:**
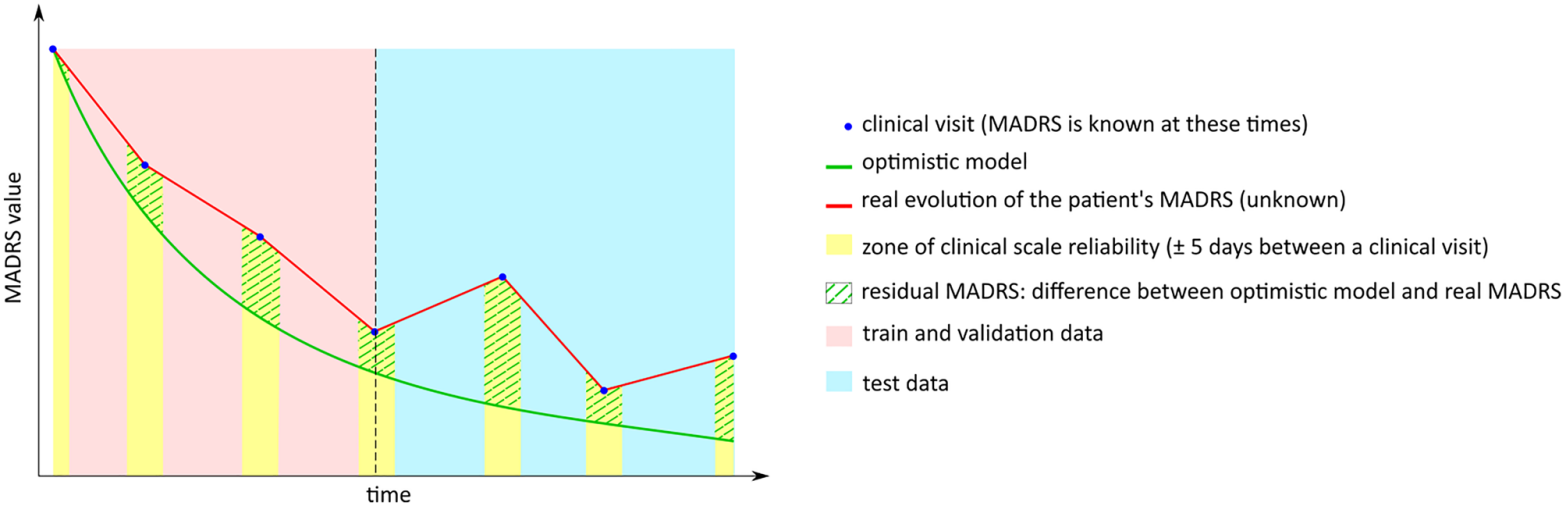
Label detrending procedure. This diagram shows how data and labels are handled and partitioned for the machine learning algorithm. The actual MADRS score is obtained only during clinical visits, although it can be safely extended to 5 days on either side of a clinical visit. The physiological data are available every day. The residual between the output of the optimistic model and the known MADRS score is used as a label for the machine learning model. The data and labels are then partitioned between train and test to fit and test the multi-layer perceptron.

To extract physiological features from sensors raw measurements, we proceeded as follows. Cardiorespiratory (such as heart rate, breathing rate, heart rate variability)^18–23^, actigraphy (e.g., L5, M10, etc.)^46,47,51^ and sleep-based physiological features (e.g., sleep stages such as REM/NREM/WASO)^52^ were extracted respectively from PPG, 3-axis accelerometer and both sensors' data using a combination of standard algorithms from the literature^53–55^. These features were further grouped into physical activity (12 features), heart rate (25 features), heart rate variability (39 features), breathing rate (12 features) and sleep (13 features) and were smoothed with a mean filter to remove potential outliers. Missing values were imputed using interpolation, and features were normalized between 0 and 1 independently of one another to account for patient heterogeneity. Due to proprietary concerns, the full list of these physiological features cannot yet be disclosed.

### Machine learning algorithm

A detailed description of the ML algorithm is available in the Supplementary Materials. The optimization procedure is divided into two parts: training SiBaMoD, which depends on hyperparameters λ and ν, respectively the recovery rate and the signature size, and an optimization scheme for selecting appropriate values of λ and ν for a given patient. SiBaMoD itself is composed of several parts: label extension and detrending processes, a feature selection, and a deep learning Multi-Layer Perceptron (MLP) model described below.

Label extension addresses the sparsity of the MADRS scores (collected once per month) relative to the abundance of physiological values (collected daily). To this end, the clinical labels were extended over a window of ± 5 days around each follow-up visit. This procedure is summarized in Fig. 1. The label extension methodology was justified by considering the test–retest reliability of the MADRS over the course of several days as described in the literature^56^ and was confirmed by experiments conducted with our dataset.

The label detrending procedure addressed the issue of having a nonstationary label over the course of the clinical trial for a given patient (since most patients tend to recover due to treatment). The detrending procedure consisted of replacing the MADRS scores with the discrepancy of an optimistic model that estimates change in the MADRS score based solely on the most recent clinical visit. This model has no trainable parameters but rather depends solely on constant *λ*. On a given day, this model predicts a MADRS score given an amelioration rate based on the previous clinical evaluation of the patient by the physician. The difference between the actual MADRS score and the MADRS score predicted by this optimistic model is called the residual MADRS score. This choice of optimistic model is supported by known models of affective disorders in the literature^57^.

The feature selection block is performed by a statistical computation on the train/validation sets. This component of the algorithm selects the *ν* most correlated features with the MADRS on the train and validation sets. To be as general as possible and to detect non-linear correlations, this component selects physiological features that minimize their independence with MADRS based on the Hilbert–Schmidt Independence Criterion (HSIC)^58^, which is more adapted to non-monotonic and non-linear signals than Spearman or linear correlation coefficients. This set of *ν* features is called the individual depression biosignature and can be used to efficiently predict the disease’s progression with respect to the clinical scale used. Thus, for each day of recorded physiological data, we can extract a subvector of dimension *ν* by selecting only the features appearing in the biosignature.

The last component of SiBaMoD is a multi-layer perceptron (MLP) which takes as input the *ν* features of the individual biosignature selected by the feature selection component, and outputs an estimate of the residual MADRS*.* Specifically, the MLP consists of an input of dimension *ν* followed by 3 hidden layers of respective dimension 8ν, 4ν, and 2ν, and a single scalar as an output. After early experiments, the parameters chosen for MLP training were batch size of 16, training for 500 epochs, and early stopping callback of 5 epochs monitoring improvements in validation loss. To smooth out random fluctuations due to kernel initialization, and to avoid having inaccurate predictions because of potential local minima in the parameter space of the model, this process is repeated 11 times and the final output prediction is set to be the median of the predictions.

**Figure 2:**
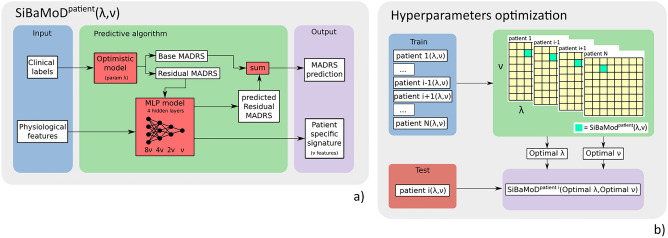
Overview of the full algorithm’s pipeline presented in this work. (**a**) The SiBaMoD pipeline with parameters (*λ*, *ν*) for a single given patient. The physiological features are used to train a multi-layer perceptron model, along with the training labels, which were detrended using the optimistic model with recovery rate *λ*. The prediction output of the model is then combined with the observed MADRS score to determine the predicted MADRS score. The *ν* features used by the model that are best correlated with the MADRS score form the patient specific signature. (**b**) In our cohort, the SiBaMoD pipeline is repeated using every patient as a test patient in a leave one patient out (LOPO) procedure, estimating the hyperparameters (*λ*, *ν*) for all patients except the test patient to determine the optimal values for these parameters.

The SiBaMoD is trained to minimize the mean square error (MSE) loss using stochastic gradient descent on the extended clinical labels (± 5 days around the follow-up visits until month 3, resulting in 30 days) with the corresponding physiological features, and is evaluated on the remaining extended clinical labels and physiological features. Once trained, SiBaMoD can be used to predict the MADRS score daily, including unlabeled days. This predicted MADRS score is then further reduced into 2 classes: healthy (MADRS score < 20) and ill (MADRS score ≥ 20) to enable the use of a binary accuracy metric to optimize the hyperparameters of the model. Details of the SiBaMoD algorithm are presented in Fig. 2a.

To determine the best λ and ν values for all patients, we optimize the binary accuracy by performing a gridsearch optimization of these parameters, using a leave one patient out (LOPO) scheme on the SiBaMoD. Specifically, the SiBaMoD pipeline is repeated such that all patients in the cohort are set as the test (left out) patient to estimate the optimal hyperparameters (λ, ν) of SiBaMoD for all other patients in the cohort. This LOPO scheme prevents overfitting; in other words, it ensures a good performance generalization across the entire pipeline for new unseen patients, even though the SiBaMoD itself is patient specific. The hyperparameter optimization scheme is presented in Fig. 2b.

**Figure 3:**
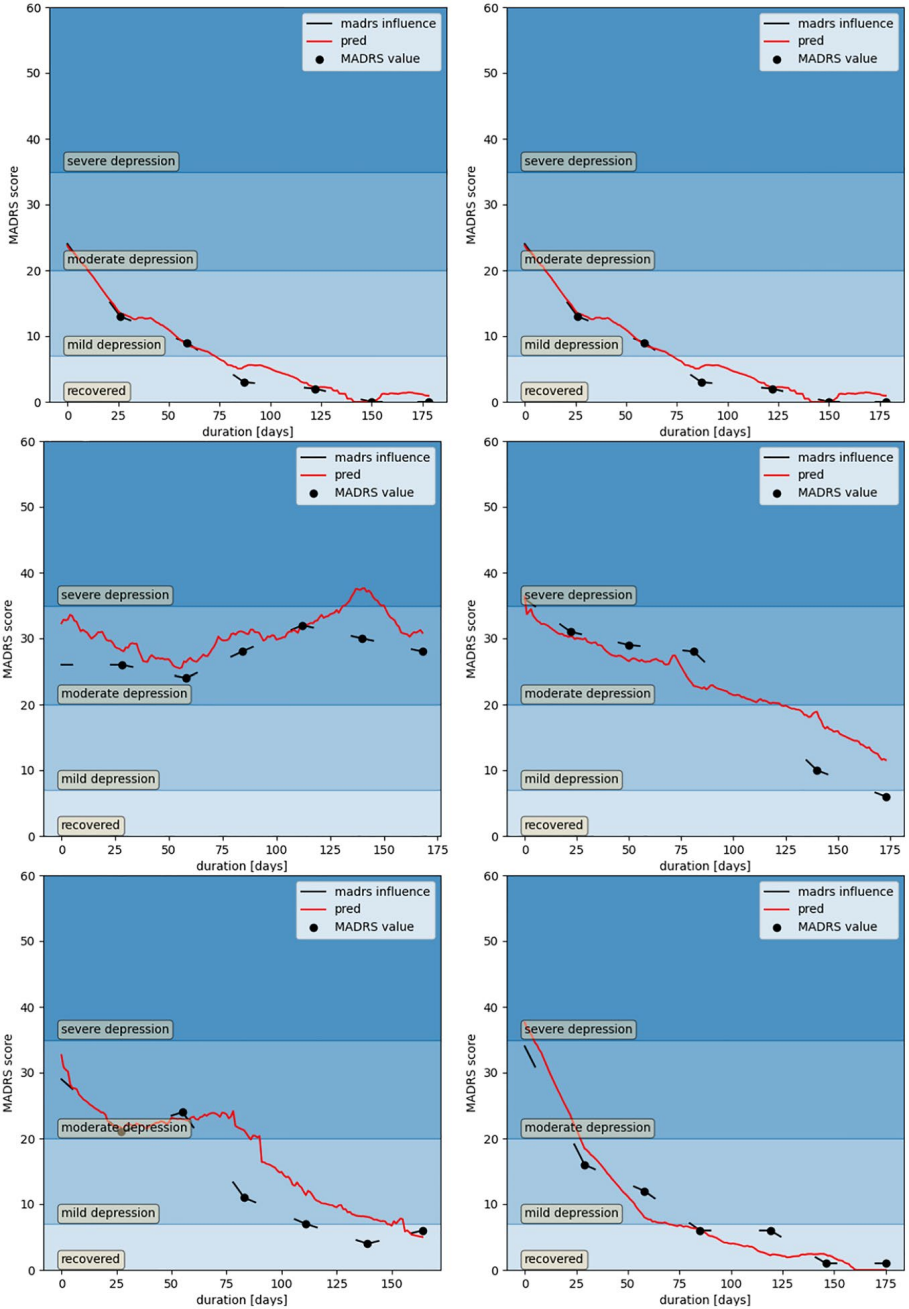
Comparison of the model's prediction with the ground truth clinical labels. Predicted MADRS evolution (in red) and actual MADRS scores measured by the physician during the monthly clinic visits (black dots) for a sample of 6 selected patients. The lines around each visit represent the extended clinical labels to 5 days on either side of a clinical visit. The background color intensity indicates the 4 classes of depression symptom severity.

Standard statistical analyses, such as analysis of variance, cannot be conducted in ML-based analyses, which are not based on a distribution of a single factor in different populations. In this work, to validate each model, we rely on the following metrics. Firstly, we compute the 2-class and 4-class accuracies of the data. Following the literature^50^, the 2 classes are obtained by merging the classes “recovered” (MADRS 0–6) with “mild depression” (MADRS 7–19), and by merging the classes “moderate depression” (MADRS 20–34) with “severe depression” (MADRS 35–60). True positive and true negative rates are reported for the binary classification task. Secondly, the mean absolute error (MAE) in MADRS, together with confidence interval with α = 0.05, are computed considering each patient as a separate sample of our true distribution. Finally, a visual inspection of the predicted curves and MADRS label (Fig. 3) given by the clinician is performed.

It should be noted that the easiest metric, namely the mean absolute (or mean squared) error in MADRS prediction is not ideal in terms of reliability and usability, since the noise in the labels themselves is important with respect to the signal we are detecting (concordance between different physicians ranging from r = 0.89 to r = 0.97^40^). However, the 2-class and 4-class classifications are more agreed upon between physicians since they are broader categories.

Considering the novelty of the database under study, we cannot directly compare our metrics to baselines from the literature, therefore we evaluate the performance of our model by comparison with 2 baseline models. The first baseline is the constant prediction model, which always predicts the majority class when classifying disease severity and the mean MADRS score in regression analyses, and the second is the optimistic model that sets the residual MADRS score to 0.

## Results

### Patient demographic and clinical characteristics

Among 40 patients enrolled, 12 patients were dropped out, 2 patients have been included in the study for less than 4 months and 26 already completed at least 4 months (out of a total of 6 months) necessary to train and test the algorithm. Amid the 12 dropped out patients, 2 had side effects (e.g., skin irritation), 2 patients were lost to monitor, and 8 patients decided to stop the clinical trial. Therefore, these 26 patients will be included in our report and its analysis post-facto. The population was 69.2% female, the average age was 50.4 ± 7.3 years, and the median age was 51 (range: 29–63) years. All patients had been diagnosed with MDD prior to inclusion. The mean number of MDD episodes at baseline was 1 ± 1.2, and the mean baseline MADRS score was 29.96 ± 5.44. Current treatment for MDD consisted of antidepressants (84.6% of patients), anxiolytics (53.8%) and psychotherapy (57.7%). These demographics and clinical characteristics are detailed in Table 1.

**Table 1:** Baseline demographic and clinical characteristics.

Variables	
Sex, M:F, n(%)	8 (30.8%): 18 (69.2%)
Mean (± SD) age, years	50.4 ± 7.3
Median (min–max) age, years	51 (29–63)
Prior MDD, yes:no:missing, n (%)	14 (53.8%):11 (42.3%):1 (3.8%)
Mean (± SD) number of previous episodes	1 ± 1.2
Median (min–max) number of previous episodes	1 (0–4)
Mean (± SD) age of first episode, years	45.8 ± 9.85
Median (min–max) age of first episode, years	47 (24–63)
Mean (± SD) baseline MADRS score	29.96 ± 5.44
Median (min–max) baseline MADRS score	29.5 (20–42)
Anxiolitic treatment, yes:no, n (%)	14 (53.8%):12 (46.2%)
Antidepressants treatment, yes:no, n (%)	22 (84.6%):4 (15.4%)
Psychotherapy, yes:no, n (%)	15 (57.7%):11 (42.3%)

The database used for the implementation of the algorithm consists of 4 to 6 months of physiological data and 5 to 7 MADRS labels for each patient. For each patient, the extended clinical labels and the corresponding physiological features up to the third month were used as a training set (totaling 30 days) and the remaining labelled data were used as test set. Slightly more than half (51.7%) of the dataset corresponded to periods when the MADRS score was ≥ 20, and the remaining data corresponded to periods when the MADRS score was ≤ 19. A total of 40.2% of the dataset corresponded to periods of time when the patients exhibited moderate depression.

### Optimal hyperparameters

**Figure 4:**
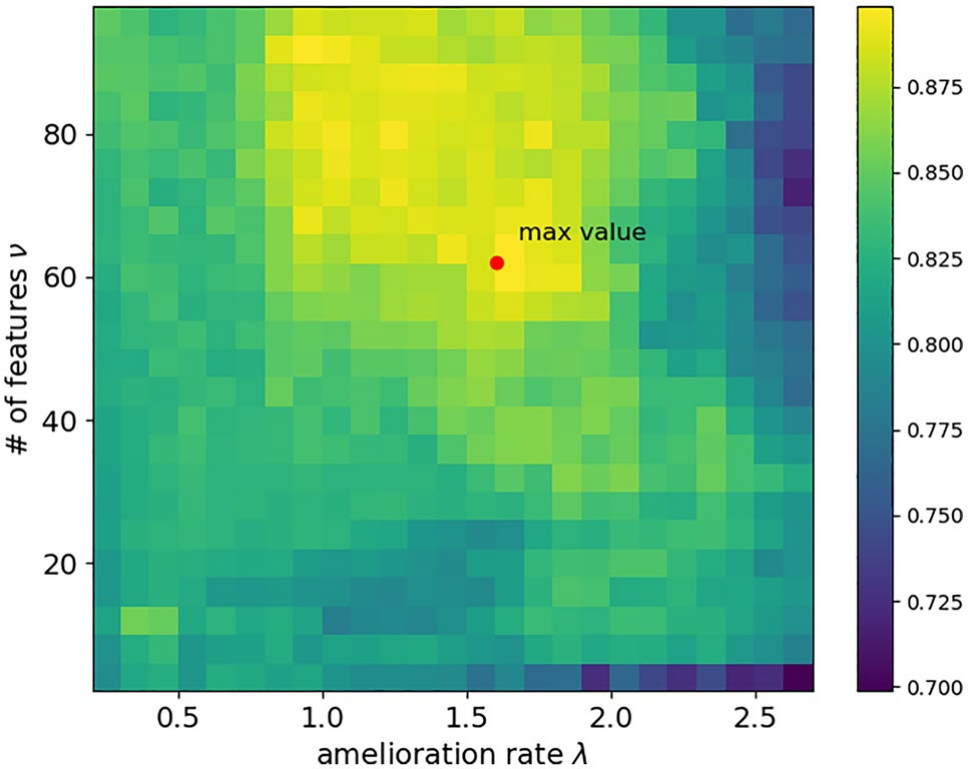
Grid search results for the choice of hyperparameters. A heatmap of binary classification accuracy with respect to the hyperparameters λ and ν for a single representative patient. The set of parameters λ = 1.6 and ν = 62 yielded to the optimal accuracy for this patient.

To determine the number of physiological features in the biosignature that optimally predicts the course of MDD in our patients via ML, we first search the hyperparameter values corresponding to this optimum. Table 2 presents the optimal hyperparameters identified for each patient using the optimization pipeline given in Fig. 2b. The number of features in the biosignature (i.e., optimal value of ν) and the selected parameter of the optimistic model (i.e., optimal value of λ) were relatively identical among the patients. An example demonstrating the evolution of the binary classification accuracy with respect to the hyperparameters λ and ν for a patient is shown in Fig. 4.

**Table 2:** Optimal values of λ and ν across patients.

Test patient number	Optimal *λ* [%]	Optimal *ν*
1	**1.6**	**62**
2	**1.6**	**62**
3	**1.6**	**62**
4	**1.6**	**62**
5	**1.6**	**62**
6	0.9	98
7	**1.6**	**62**
8	**1.6**	**62**
9	**1.6**	**62**
10	**1.6**	**62**
11	0.8	90
12	**1.6**	**62**
14	**1.6**	**62**
15	**1.6**	**62**
16	**1.6**	**62**
17	1.7	78
18	**1.6**	**62**
19	**1.6**	**62**
20	1.2	68
21	**1.6**	**62**
22	**1.6**	**62**
23	**1.6**	**62**
24	**1.6**	**62**
25	**1.6**	**62**
26	**1.6**	**62**

The value of the hyperparameters λ and ν that maximized the binary classification accuracy for all patients but the test patient (in the leave one patient out method) is selected by a grid search. Strong stability is observed among the patients in both parameters. The most frequent stable values are shown in bold.

### Prediction performances

Our model achieved 63.2% accuracy in the 4-class severity classification task and 86.0% accuracy in the 2-class severity classification task, the latter of which was 11.6% above the baseline prediction and 34.3% above the constant prediction of the majority class. For the raw MADRS prediction, the model reported a mean absolute error (MAE) of 6.7 (95% CI 3.4–10.1) from the actual MADRS score.

**Figure 5:**
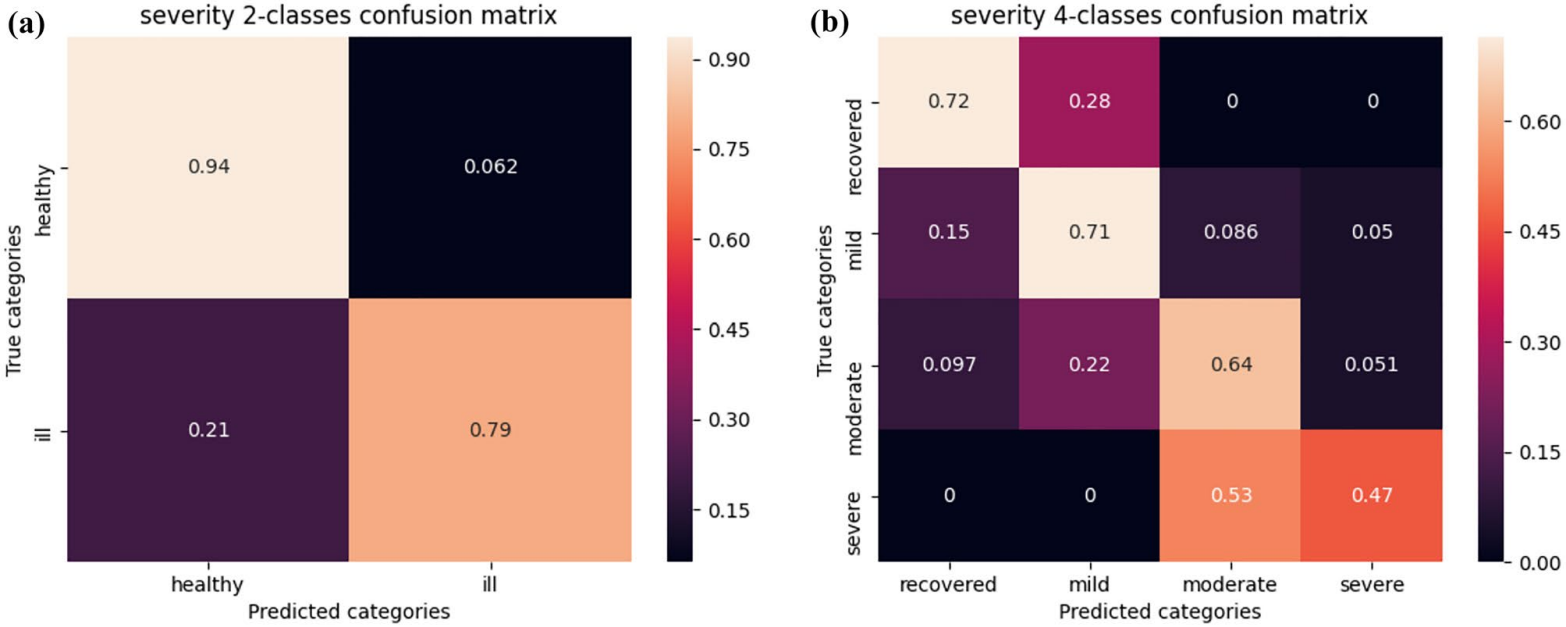
Confusion matrices of our categorical predictions. (**a**) Left: Normalized confusion matrix of our model’s 2-class prediction for the test set. The model had a true negative rate of 94%, which can be explained by the tendency of the optimistic model to output a recovery prediction. (**b**) Right: Normalized confusion matrix of our model’s 4-class prediction for the test set. Errors far from the diagonal are zero or close to zero. Notably, our model never misclassifies recovered and severe depression or vice versa.

We then chose to represent the performance of the model in the classification tasks in the form of confusion matrices. The normalized confusion matrices of the predictions in the 2-class (depressed/not depressed) and 4-class (recovered/mild depression/moderate depression/severe depression) contexts are shown in Fig. 5. A perfect prediction corresponds to a matrix with ones along the diagonal. For 2-class prediction, the sensitivity (i.e., true positive rate, TPR) was 79%, and the specificity (i.e., true negative rate, TNR) was 94%. For the 4-class prediction, there was no confusion between severe depression and recovered, and low confusion between the recovered and other classes. Overall, the model outperforms both baselines, and its TPR is lower than its TNR. These results are detailed in Table 3. Even though the model is trained on extended clinical labels, it gives a daily MADRS score prediction provided that physiological features are available. Example of resulting prediction curves are displayed along with the extended clinical labels in Fig. 3.

**Table 3:** Comparison between our model and two baselines.

Method	2-class accuracy (TPR; TNR)	Predicted MADRS MAE
Constant (baseline) model	51.7%	9.63 (95% CI 7.83–11.43)
Optimistic model (prediction without physiological data)	74.4% (33.3%; 97.8%)	7.21 (95% CI 4.7–9.7)
Our model (baseline + deep learning approach)	**86% (79%; 94%)**	**6.7 (95% CI 3.4–10.1)**

Perturbation study of our model. The 2-classes test accuracy was computed for the discrimination between ‘ill’ and ‘healthy' classes. The true positive rate (TPR) and true negative rate (TNR) are also reported. The predicted MADRS MAE refers to the mean absolute error (95% confidence interval) in MADRS scores estimated by the model on the test set. The best results are reported in bold font and correspond to our deep learning approach.

### Biosignature characteristics

**Figure 6:**
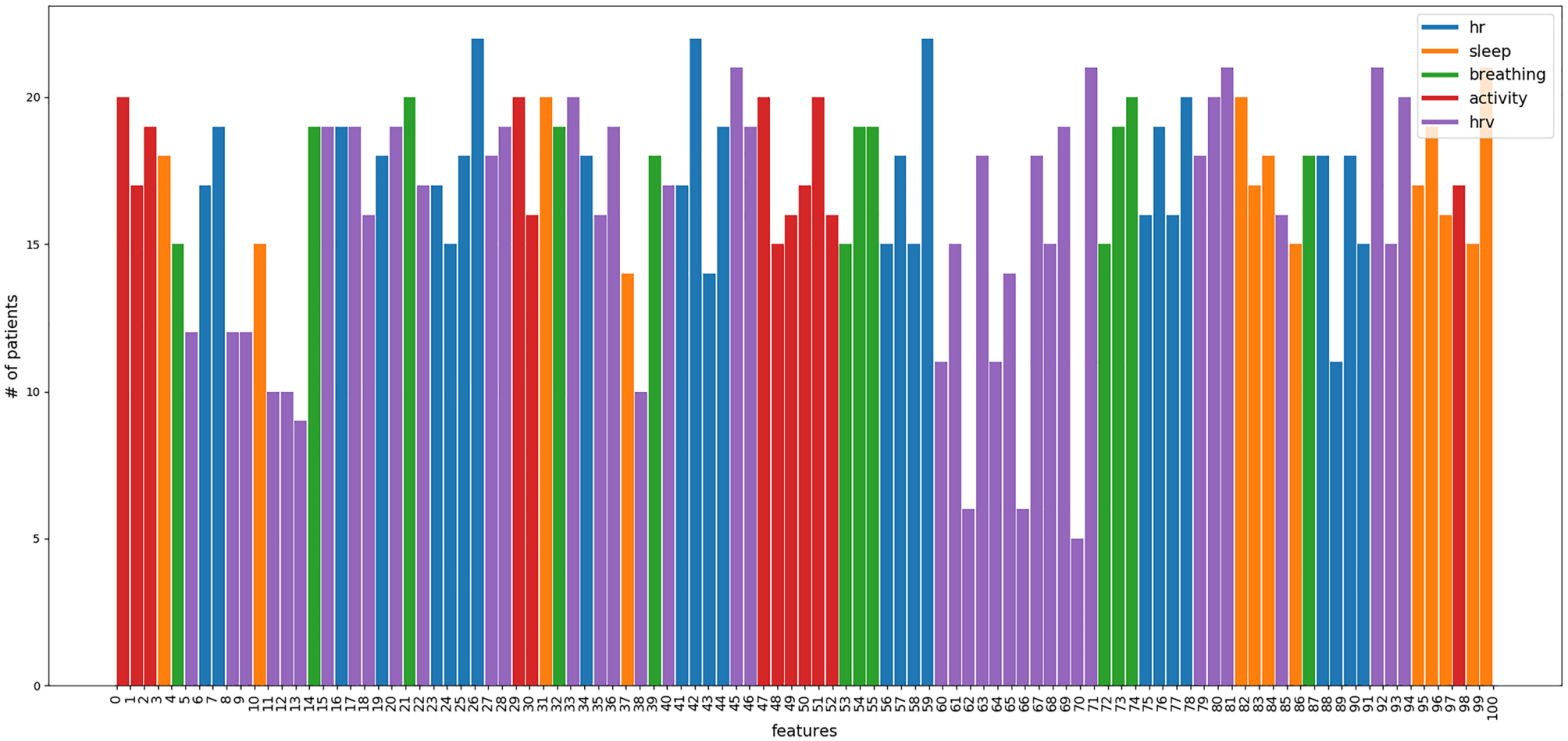
Histogram of features apparition in biosignatures. Histogram of unique features included in the biosignature of each patient. No feature was selected only for a single patient, and no feature was selected for all patients in the cohort. The colors represent the feature group to which the physiological variable belongs.

The biosignature being the set of physiological variables used in the model's prediction and considering the predictive power of the ML model as demonstrated in the abovementioned accuracies, it can be interpreted as a presentation of each patient's MDD symptoms. Further analysis indicated that each patient had a unique signature composed of multiple physiological features (Fig. 6); no feature appeared in all patients at once, and no feature appeared solely in the biosignature of a single patient, indicating that the model correctly captured the physiological manifestation of symptoms. The individual biosignature detected by our system is composed of 62 features, except for patients 6, 11, 17, 20 which have 98, 90, 78, and 68 features respectively (Table 2).

**Figure 7:**
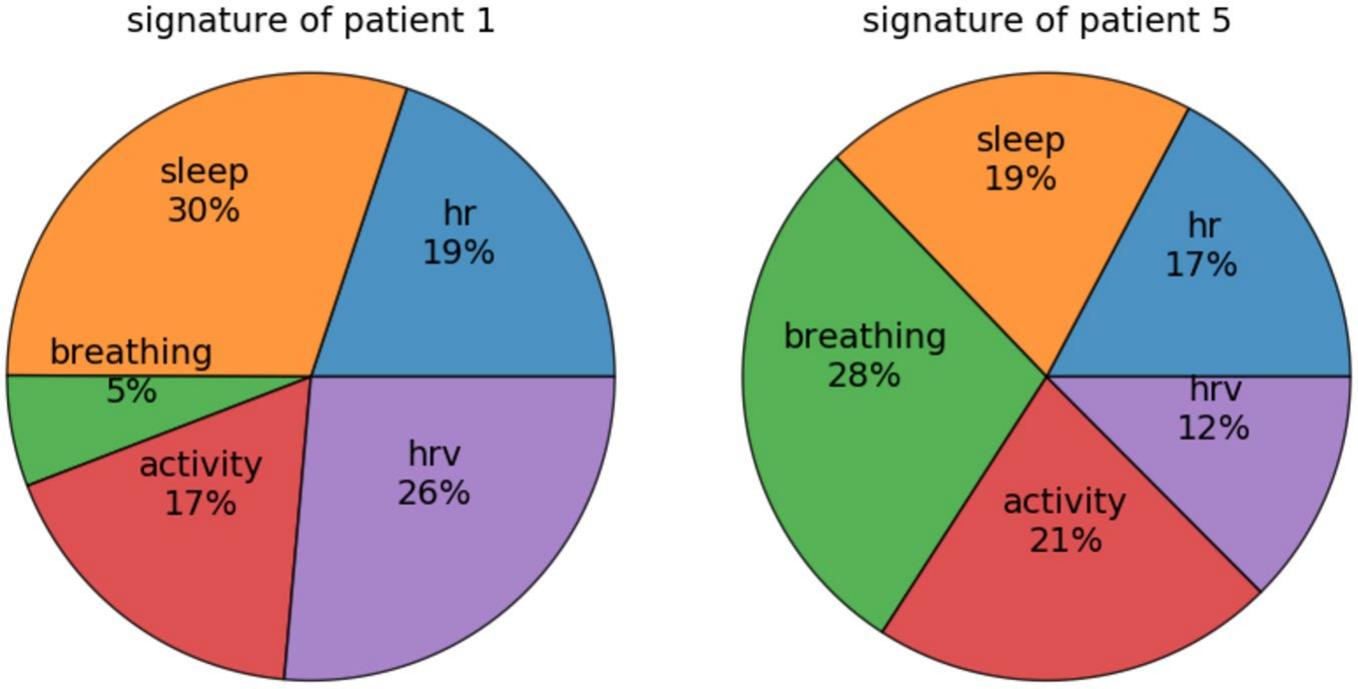
Physiological features repartition in feature groups for 2 patients. Example biosignatures of depressive symptoms determined by our algorithm for 2 patients (see Table 2 for their optimal features number ν). The circular histogram represents the importance of each group of features in the patient’s signature; a group is considered more important if the size of its wedge is larger. The symptoms of patient 1 are more represented in the sleep variables, whereas those of patient 5 are visible in the breathing rate variables.

Furthermore, the biosignature can be individually investigated. For readability, we can group the physiological features into 5 clusters according to their physiological relevance: heart rate, heart rate variability, sleep, breathing, and activity, and count the number of features appearing in each group. For instance, a biosignature almost entirely composed of sleep-related physiological features means that the corresponding patient’s symptom is mostly expressed through sleep disturbances. Figure 7 shows two examples biosignatures in terms of the categories of physiological features included.

## Discussion

This work has validated the hypothesis that a supervised ML system can efficiently predict a patient’s clinical score by identifying their biosignature of symptoms during a MDD episode. We observed that, after training with 3 months of data, the ML algorithm could predict patient mood status over the next 3 months with good accuracy. The algorithm was trained with multiple physiological features collected by means of a wearable device from 26 outpatients with MDD (MADRS score ≥ 20) who participated in a 6-month prospective multicenter study. The sex distribution in our cohort (69.2% female) is consistent with the worldwide distribution of MDD, with a higher prevalence of depressive disorders among women than men (range 1.3–3.1:1, unadjusted mean sex ratio: 2.1:1 for lifetime, 1.7:1 for point prevalence rates^3^).

Despite the reasonably stable number of physiological features included in each signature, each patient had a unique biosignature consisting of different features, which highlights the value of a multimodal approach that encompasses numerous physiological metrics that may differ between patients. This finding aligns with the known heterogeneity of MDD manifestations^9^. Our ML model accounted for each patient’s deviation from the optimistic model according to their physiological data. While the accuracy of the optimistic model was 74.4%, our entire deep learning model achieved an accuracy of 86.0%. This confirms that the phenotypic expression of depression can be observed in physiological variables.

This was the first cohort to be extensively screened with multimodal approaches (i.e., by collecting data on multiple physiological dimensions such as cardiorespiratory and physical activity signals) with close patient follow-up for 6 months. Although the optimistic model achieved good results, by design it tends to predict recovery and thus performs poorly in patients with a poor response to treatment or who experience relapses (TPR 33.3%). Adding deep learning to this baseline model allowed us to achieve better performance and reach a TPR of 79% for disease detection, even in patients with worse outcomes. To our knowledge, no study published to date has presented a model of MDD evolution validated on the MADRS score of individual patients, consequently, a direct comparison of the metrics obtained by our model with results from other studies is not applicable.

Most patients showed optimal hyperparameters at *λ* = 1.6% and *ν* = 62; however, those identified for patients 6, 11, 17, and 20 were beyond the mode values. Since the hyperparameters for a given patient are determined by maximizing performance for all patients except the test patient (patients 6, 11, 17, or 20 respectively), these four patients were essential to ensure the presence of patient diversity and disease phenotype variation within the cohort. In other words, excluding data from these patients would decrease the diversity of the dataset and potentially make it impossible to accurately determine the optimal values of hyperparameters. To estimate an upper bound for the model’s performance on a larger cohort, we tested the accuracy of a model trained on a global choice of hyperparameters, optimizing the average accuracy for the full cohort of patients (i.e., without the LOPO scheme). In this case, our model achieved an average accuracy of 92.6%. However, this procedure suffers from data leakage since test data from a patient were used in the training process to estimate the hyperparameters of the optimistic model. This experiment shows that the model’s performance is limited by patient heterogeneity. Interestingly, this phenomenon can be observed in an objective and explicit manner by observing the diverse patient biosignatures yielded by our approach. More generally, this biosignature may be able to categorize all expressions of depression, known and unknown. This signature provides supplementary objective observations that clinicians can use to assess their patients, with the aim of eventually incorporating other information (such as self-report data, personal feelings and history) to reinforce the practitioner’s diagnosis.

Our study has some limitations. The sample size (26 analyzed patients, 3 months of training data per patient) was modest, although it provided sufficient power to construct our ML model and achieve good predictive performance. For this reason, we had to mitigate the risk of overfitting by implementing multiple strategies: the feature selection component reduces the input dimensionality of our model, the model itself is shallow, and early stopping was added to regularize it. Generalization of baseline algorithms can be improved by including patients with more varied responses to treatment (e.g., patients with relapse after a few months) and by developing a baseline model that would incorporate more diverse modeling of the disease, thus improving the general performance of the whole model. Moreover, as discussed above, the optimistic model tends to predict recovery, but adding a deep learning model mitigated this tendency. To improve further relapse detection, and more generally the sensitivity of our model, we should replace our optimistic model by a more complex model of the disease’s clinical evolution. Other limitations are due to the novelty of this work. It is impossible to compare our findings with another study and to replicate the experiments on another dataset, potentially leading to an overestimation of the model’s performance. However, our study is multicentric, and our training used a LOPO scheme, mitigating these limitations. Replicating the study on a dataset collected with another device could also strengthen our results and validate the performance of our model.

In conclusion, this study presents an ML-based approach that allows for the development of an individual predictive biosignature of MDD based on various physiological features obtained from passive sensors. In future clinical applications, psychiatrists could use this work to get access to a daily physiological assessment of their patients, allowing a better follow-up and therefore detect mood status deterioration earlier and establish more informed diagnosis. Specifically, clinicians will detect a relapse on the day it occurs, instead of 15 days late in average for monthly visits. Moreover, our model’s true negative rate of 94% will not increase the burden of false detection, but instead strengthen the physician’s diagnosis since its overall true positive rate of 79% is higher than the misdiagnosis rates reported in clinical studies^59–61^. Furthermore, this biosignature could be analyzed to identify the most important symptoms experienced by a patient, regardless of whether the patient is aware of these symptoms or has reported them to the physician. The biological signature of depression generated by SiBaMoD could also provide new avenues of research; for instance, a clustering analysis of the biological signatures of a number of patients may allow for a new categorization of MDD phenotypes based on objective markers, which could lead to the development of precision medicine in psychiatry.

## Supplementary Information

Supplementary Information.

## Data Availability

The data are not publicly available due to privacy and ethical restrictions regarding patient privacy protection policies. Please contact the corresponding author for all inquiries concerning data.
