# Supplementary material for: Predictive biosignature of major depressive disorder derived from physiological measurements of outpatients using machine learning
Source: Sci Rep. 2023 Apr 25;13:6332. doi: 10.1038/s41598-023-33359-w (PMC10130089; doi:10.1038/s41598-023-33359-w)
Supplement: Supplementary file 1 — Supplementary Information. [file 41598_2023_33359_MOESM1_ESM.pdf]

Predictive biosignature in Major Depressive Disorder from ambulatory physiological measurements using machine learning-based algorithms

Nicolas RICKA[1,*], Gauthier PELLEGRIN[1], Denis A. FOMPEYRINE[1], Bertrand LAHUTTE[6], and Pierre A. GEOFFROY[2,3,4,5]

[1]MyndBlue, F-75008 Paris, France
[2]Psychiatry and Addictology Service, AP-HP, GHU Paris Nord, DMU Neurosciences, Hopital Bichat - Claude Bernard, F-75018 Paris, France
[3]GHU Paris - Psychiatry & Neurosciences, 1 rue Cabanis, F-75014 Paris, France
[4]Université de Paris, NeuroDiderot, Inserm, FHU I2-D2, F-75019 Paris, France
[5]CNRS UPR 3212, Institute for Cellular and Integrative Neurosciences, F-67000, Strasbourg, France
[6]Chief of Psychiatry Department, Bégin Military Hospital, F-94160 Saint-Mandé, France
[*]Corresponding author: nicolas@myndblue.ai

Supplementary data

Machine learning algorithm

Overview

Our dataset is composed, for each patient p, of 6 months of daily physiological features $\mathbf{x_d^p}$ together with 7 MADRS scores corresponding to 7 follow-up visits at days $d_0, d_1, \ldots d_7$. In order to be able to use this dataset with our algorithms, it was necessary to extend the MADRS labels to achieve correspondence between the physiological features and MADRS score corresponding to this specific day. To this end, clinical labels were extended over a window of ± 5 days around each follow-up visit, assuming that the MADRS is sufficiently stable such that each day from $d_i - 5$ to $d_i + 5$ can be associated with a MADRS score interpolated from MADRS between days d_{i-1},d_i and d_i, d_{i+1}.This whole procedure is summarized in Figure 2.

The procedure can be justified by considering the test-retest reliability of the MADRS over the course of several days as described in the literature [1] and was confirmed by experiments conducted on our dataset. The 5-day window also corresponds to the optimal trade-off in terms of data quantity versus data/label relationship stability. Thus, except when explicitly stated otherwise, each pair (x, y) of physiological features and MADRS score corresponds to the daily physiological measures and associated MADRS score at a day that is at most 5 days apart from a follow-up visit. Since physiological data is available daily for the patients (and not only ± 5 days around the follow-up visit), the full physiological time series can be used for visual inspection of the model's prediction. However, these other physiological measurements will not be used to train or evaluate the model since it is not possible to associate a ground truth MADRS score corresponding to these days.

The ML model has been constructed for the following clinical situation: given a cohort P of train patients, and the data corresponding to daily physiological features and first 4 follow-up visits (baseline, M1 to M3) of the test patient p, predict the clinical scales of the test patient over the remaining labelled daily data (from the follow-up visit at day d_4 up to day d_7 if available). Consequently, the train/validation datasets are given as

$$X_{train}, Y_{train} = \{(X^p_{d<d_4}, Y^p_{d<d_4})\} \cup \{(X^{p'}, Y^{p'})\ \forall p' \in P\},$$

where every data points $(\mathbf{x}, y)$ consists of the physiological measures at a given day d and its associated MADRS score y (for d at distance less than 5 days from a follow-up visit). The training/validation dataset contains data coming from $P+1$ patients in total: the full data of P patients plus the datapoints between d_0 and d_4 of the test patient. The test set of our pipeline is:

$$X_{test}, Y_{test} = (X^p_{d \geq d_4}, Y^p_{d \geq d_4}).$$

Our ML algorithm has 2 optimization loops. For each patient, a Signature Based Model of Depression (SiBaMoD) is defined (described in the subsection Signature Based Model of Depression). The SiBaMoD depends on two key hyperparameters, λ and ν which are also described later. Given these hyperparameters, SiBaMoD(λ, ν) takes as input a day of physiological features $\mathbf{x}$ and outputs a MADRS score estimation y. For any patient of the dataset, this intra-patient model is trained on $\{(X_{d<d_4}, Y_{d<d_4})\}$, and is evaluated on $\{(X_{d\geq d_4}, Y_{d\geq d_4})\}$.

To avoid data leakage from the test patient p, the choice of hyperparameters λ, ν is performed by an optimization loop on the train cohort P. Explicitly, a single SiBaMoD(λ, ν) is trained for each patient p' in P, and hyperparameter values are selected that maximize the binary classification accuracy averaged on all train patients p'. Once the hyperparameters are selected, a single SiBaMoD is trained on $\{(X^p_{d<d_4}, Y^p_{d<d_4})\}$, *i.e.* the training data of the test patient, and the performance metrics are computed on the test set $X_{test}, Y_{test} = (X^p_{d\geq d_4}, Y^p_{d\geq d_4})$. These optimization loops are summarized in Figure 3.

Standard statistical analyses, such as analysis of variance, cannot be conducted in ML-based analyses, which are not based on a distribution of a single factor in different populations. Classification is typically measured either by confusion matrices, through a comparison of the classification accuracy on the test set, or in the case of regression by using an error metric such as the mean absolute error (MAE). Since each patient has only a few datapoints in their test set, classification metrics are averaged using a leave one patient out test scheme.

In this work, to validate each model, we rely on the following metrics. Firstly, the 2-class and 4-class accuracies of the data. The 2 classes are obtained by merging the classes 'recovered' (MADRS 0-6) with 'mild depression' (MADRS 7-19), and by merging the classes 'moderate depression' (MADRS 20-34) with 'severe depression' (MADRS 35-60), as described in previous works [2]. True positive and true negative rates are reported for the binary classification task. Secondly, the mean absolute error (MAE) in MADRS, together with confidence interval with $\alpha = 0.05$, are computed considering each patient as a separate sample of our true distribution. Finally, a visual inspection of the predicted curves and MADRS label given by the clinician is performed.

It should be noted that the easiest metric, namely the mean absolute (or mean squared) error in MADRS score prediction is not ideal in terms of reliability and usability, since the noise in the labels themselves is important with respect to the signal we are detecting (concordance between different physicians ranging from r = 0.89 to r = 0.97 [3]). However, the 2-class and 4-class classifications are more agreed upon between physicians, since they are broader categories.

Signature Based Model of Depression (SiBaMoD)

The main technical part of our algorithm, the SiBaMoD, depends on 2 hyperparameters, a real number $\lambda > 0$ and a positive integer ν described below. When there is a risk of ambiguity, this will be denoted by SiBaMoD(λ, ν) to make the hyperparameters explicit. These hyperparameters are fixed throughout this section, and the reader can refer to the next section for the selection method of these hyperparameters. The SiBaMoD is patient specific: for each patient p, there is a different model SiBaMoD$^p(\lambda, \nu)$. For each day d in patient p's data, this ML model predicts an estimate of the MADRS score of patient p at day d from the physiological measurements of this same patient p at day d.

SiBaMoD$^p(\lambda, \nu)$ is divided into 3 main components: a feature selection block, an optimistic model, and a multi-layer perceptron (MLP). To perform a prediction, $\hat{y}^p_d$ from $\mathbf{x^p_d}$, SiBaMoD first have to learn the specific relationship between physiological measures and the clinical scale of patient p's depressive symptoms. To this end, patient p's datapoints are divided into a training/validation set: $\{(X^p_{d<d_4}, Y^p_{d<d_4})\}$, further decomposed into a training set (first 80%) and validation set (last 20%), and a test set $\{(X^p_{d\geq d_4}, Y^p_{d\geq d_4})\}$. The training set is used to parameterize the model, validation is used to control overfitting, and the test set is only used to compute the metrics and evaluate the model's performance.

We now turn to describing the 3 components of SiBaMoD and their interaction. Again, we emphasize that, except when specifically stated otherwise, this subsection follows the convention described above: all datapoints considered here correspond to days that are at most 5 days apart from a clinical evaluation. The reader can refer to Figure 3a in parallel to this description.

The feature selection block is performed by a statistical computation on the train/validation sets. This component of the algorithm selects the ν most correlated features with the MADRS on the train and validation sets. To be as general as possible and to detect non-linear correlations, this component selects physiological features that minimize their independence with MADRS based on the Hilbert-Schmidt Independence Criterion (HSIC) [4], which is more adapted to non-monotonic and non-linear signals than, for instance, the Spearman correlation. This set of ν features is called the depression biosignature of patient p, as it is dependent on the patient, and can be used to efficiently predict the disease's progression with respect to the clinical scale used. Thus, for each day d of recorded physiological data, we can extract a subvector of dimension ν, say $\bar{\mathbf{x}}^{\mathbf{p}}_{\mathbf{d}}$ by selecting only the features appearing in the biosignature of patient p.

The optimistic model is a baseline prediction of MADRS evolution based on the most recent clinical follow-up visit. It does not have any trainable parameters, but depends solely on the fixed hyperparameter λ. On day d, the optimistic model predicts a MADRS score of

$$y_{d_i} * \exp(-\tilde{\lambda} * (d - d_i)),$$

where d_i is the previous clinical evaluation of patient p by their physician. In other words, the optimistic model predicts a daily amelioration rate of λ , where $\tilde{\lambda} = -\ln(1-\lambda)$. From now on, the parameter λ will be expressed as a daily amelioration percentage. The difference between the actual MADRS score and the MADRS score expected by this optimistic model is called the residual MADRS score. The choice of a decreasing exponential model for the optimistic model is supported by known models of affective disorders in the literature [5].

The last component of SiBaMoD$^p(\lambda, \nu)$ is an MLP which takes as input the set of ν features in the biosignature of patient p selected by the feature selection component, and outputs an estimate of the residual MADRS score from the physiological features of patient p that appear in their biosignature, that is $\bar{\mathbf{x}}_{\mathbf{d}}^{\mathbf{p}}$. Specifically, the MLP consists of an input of dimension ν followed by 3 hidden layers of respectively 8ν, 4ν and 2ν neurons, and an output of dimension 1 which is the scalar prediction for $\bar{y}^p$. After early experiments, the parameters chosen for MLP training were batch size of 16, training for 500 epochs, and early stopping callback of 5 epochs monitoring improvements in validation loss.

This model is trained on the train dataset, and validated on the validation dataset defined previously. To smooth out random fluctuations due to kernel initialization, and to avoid having inaccurate predictions because of potential local minima in the parameter space of the model, this process is repeated 11 times and the final output prediction is set to be the median of the predictions.

Global fit of hyperparameters

The procedure described here aims at training an ML model that extract a biosignature of depressive symptoms, and an estimation of the MADRS score for each incoming patient. Considering the small size of our dataset and the high risk of overfitting, extra-attention was made to avoid information leakage: the last 3 months of the test patient only appears when testing the model and was not used in any way prior to that. The procedure is depicted in Figure 3. This entire procedure is repeated for each patient as test patient. For a fixed test patient p, as described in the Overview, the selection of the hyperparameters $\lambda^p_{optimal}$ and $\nu^p_{optimal}$ to use for SiBaMoD$^p(\lambda^p_{optimal}, \nu^p_{optimal})$ is determined on the set of train patients p'. Explicitly, we train a SiBaMoD$^{p'}(\lambda, \nu)$ for all patients $p' \neq p$ of our cohort for each $0.2 \leq \lambda \leq 2.7$ and each $2 \leq \nu \leq 101$, and report the average binary classification accuracy over patients in the train cohort, of each pair (λ, ν). We then define

$$\lambda^p_{optimal}, \nu^p_{optimal} = \text{argmax}_{\lambda,\nu}\text{mean}_{p' \neq p}(\text{binary accuracy}(\text{SiBaMoD}^p(\lambda, \nu))).$$

References

1. Rykov, Y., Thach, T.-Q., Bojic, I., Christopoulos, G. & Car, J. Digital biomarkers for depression screening with wearable devices: Cross-sectional study with machine learning modeling. *JMIR mhealth uhealth* **9**, DOI: 10.2196/24872 (2021).
2. Snaith, R. P., Harrop, F. M., Newby, D. A. & Teale, C. Grade scores of the montgomery—Åsberg depression and the clinical anxiety scales. *Br. J. Psychiatry* **148**, 599–601, DOI: 10.1192/bjp.148.5.599 (1986).
3. Li, X., Zhang, X., Zhu, J., Mao, W., Sun, S., Wang, Z. *et al.* Depression recognition using machine learning methods with different feature generation strategies. *Artif. Intell. Med.* **99**, DOI: 10.1016/j.artmed.2019.07.004 (2019).
4. Gretton, A., Fukumizu, K., Teo, C. H., Song, L., Schölkopf, B. & Smola, A. A kernel statistical test of independence. In *Advances in Neural Information Processing Systems* (2007).
5. Guizzaro, L., Morgan, D., Falco, A. & Gallo, C. Hamilton and madrs scales are interchangeable in meta-analyses but can strongly disagree at trial-level. *J. Clin. Epidemiol.* **124**, DOI: 10.1016/j.jclinepi.2020.04.022 (2020).

Figures

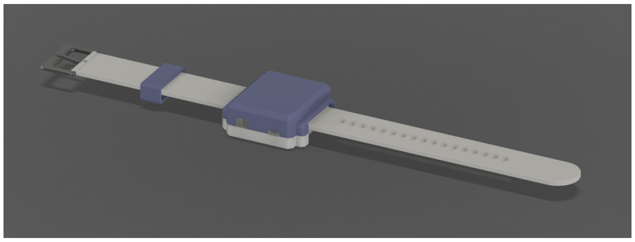

Figure 1. The wristband used for data collection throughout the clinical trial.

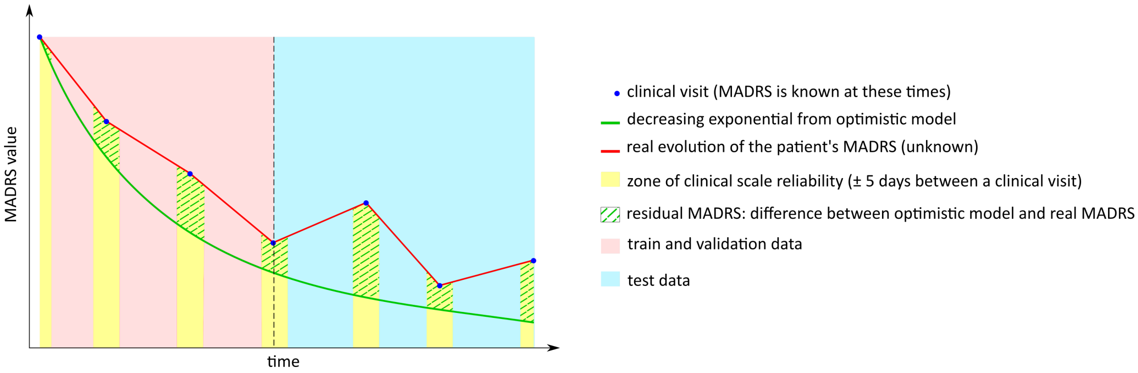

Figure 2. Label detrending procedure. This diagram shows how data and labels are handled and partitioned for the machine learning algorithm. The actual MADRS score is obtained only during clinical follow-up visits, although it can be safely extended to 5 days on either side of a clinical follow-up visit. The physiological data are available every day. The residual between the output of the optimistic model and the known MADRS score is used as a label for the machine learning model. The data and labels are then partitioned between train and test to fit and test the multi-layer perceptron.

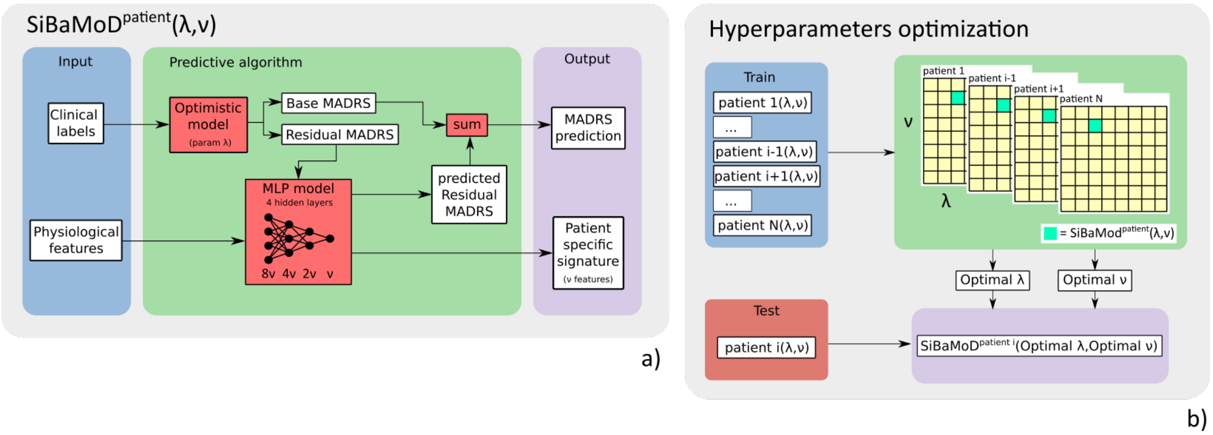

Figure 3. Overview of the full algorithm's pipeline presented in this work.
a) The SiBaMoD pipeline with parameters (λ, ν) for a single given patient. The physiological features are used to train a multi-layer perceptron model, along with the training labels, which were detrended using the optimistic model with recovery rate λ. The prediction output of the model is then combined with the observed MADRS score to determine the predicted MADRS score. The ν features used by the model that are best correlated with the MADRS score form the patient specific signature. **b)** In our cohort, the SiBaMoD pipeline is repeated for all patients as a test patient in a leave one patient out (LOPO) procedure, estimating the hyperparameters (λ, ν) for all patients except the test patient to determine the optimal values for these parameters.
